# The impact of couple-focused counseling based on the Ex-PLISSIT model on the sexual self-concept of infertile couples: a randomized trial

**DOI:** 10.1038/s41598-026-43203-6

**Published:** 2026-05-07

**Authors:** Raziyeh Dalvand, Marziyeh Otogara, Arezoo Shayan, Farideh Kazemi, Mohammad Haghighi

**Affiliations:** 1 Mother and Child Care Research Center, Institute of Health Sciences and Technologies, Hamadan University of Medical Sciences, Hamadan, Iran; 2 Department of Psychiatry, School of Medicine, Behavioral Disorders and Substance Abuse Research Center, Institute of Mental Health and Addiction, Sina (Farshchian) Educational and Medical Center Hamadan University of Medical Sciences, Hamadan, Iran

**Keywords:** Sexual self-concept, Couple-centered counseling, Infertility, Ex-PLISSIT model, Randomized trial, Psychology, Human behaviour

## Abstract

The negative impact of infertility and its treatments on sexual health is well-documented. Self-concept plays a crucial role in sexual behavior and overall sexual health. This study aims to investigate how couple-oriented counseling, using the Ex-Plissit model, can influence the sexual self-concept of infertile couples. This parallel-group randomized controlled clinical trial was conducted in Hamadan, Iran, at the infertility clinic of Fatemieh Hospital, from 2022 to 2023. One hundred twenty individuals (60 infertile couples) who referred to the clinic were randomly assigned to either the intervention group (*n* = 30 couples) or the control group (*n* = 30 couples). Based on entry criteria, the allocation sequence was determined using six blocks of 4 before the study began. Demographic characteristics and a self-reported sexual self-concept questionnaire were used to identify infertile couples with negative sexual self-concepts. The intervention group received four sessions of couple-centered sexual counseling based on the Ex_PLISSIT model, while the control group received routine treatments. Registration date in IRCT: 2022-09-13 .After the sessions, both groups completed the sexual self-concept questionnaire again. Data analysis was performed using SPSS version 26 software, including descriptive and inferential statistics (paired T-test, independent T-test, Wilcoxon, covariance) with a significance level of *P* < 0.05. The research findings indicated that the average score of women’s sexual self-concept in the intervention group before the intervention was 21.50 (4.86), slightly higher than the control group’s average of 19.87 (8.41). However, this difference was not found to be statistically significant (*p* = 0.361). Furthermore, there was no significant difference in the average sexual self-concept scores between the intervention and control groups of women after the intervention (*p* = 0.053). Interestingly, there was a statistically significant difference in the average sexual self-concept scores between the intervention and control groups of men after the intervention (*p* = 0.003). The paired t-test results revealed a statistically significant difference in the average score of men’s self-concept before and after the intervention group intervention (*p* = 0.001). Furthermore, when comparing the mean (standard deviation) of men’s self-concept scores in the post-intervention phase while controlling for pre-intervention scores, smoking, and male occupation, the average score in the intervention group was found to be 17.45 (3.23) lower than the control group’s average score of 21 (3.23). This difference was also statistically significant (*p* = 0.001). The results of the current study indicate that couple-based counseling using the Ex-PLISSIT model has a significant impact on improving negative sexual self-perception in infertile couples. Therefore, infertile couples visiting fertility centers should be evaluated for their sexual function and self-perception. If needed, they should be offered educational, therapeutic, and counseling programs to provide them with the necessary information.

Trial registration: Iranian Registry of Clinical Trial IRCT20120215009014N443.

## Introduction

Fertility and reproduction are fundamental aspects of human existence^1,2^. The challenge of infertility has long been a significant issue impacting people’s lives, causing adverse effects. Throughout history, couples have faced difficulties in reproducing, leading to ongoing struggles with infertility^3^. Infertility is a condition where couples are unable to conceive after a year of regular unprotected sexual intercourse, and it can be attributed to either partner^4^. The World Health Organization (WHO) estimates that infertility affects around 9–12% of the global population, equating to approximately 186 million individuals^5^. In Iran, the prevalence of infertility is reported to be 13.2%^6^. A recent meta-analysis (2023) estimates the prevalence of lifetime infertility in Iran at 10.9% and current infertility at 3.3%, with a slight increase from previous estimates, highlighting the need for targeted interventions in regions like Hamadan^7^. Researchers have reported that social, emotional, and psychological disorders resulting from infertility can lead to sexual and marital problems between couples^2^. The evaluation and treatment of infertility can result in effects such as turmoil, frustration, worthlessness, feelings of inadequacy, rejection, and failure in life^8^.

Self-concept is a broad, multifaceted construct that encompasses an individual’s overall perceptions, beliefs, and evaluations of themselves across various dimensions, including physical, emotional, social, spiritual, and other aspects that shape personal identity^8^.

Sexual self-concept, as a specific and critical subdomain of overall self-concept, refers to an individual’s cognitive and affective perceptions of themselves as sexual beings. It includes aspects such as sexual identity, sexual competence, sexual desirability, sexual anxiety, sexual monitoring, and beliefs about sexual behaviors and relationships^8,9^. In the Iranian context, where cultural, religious, and societal norms strongly emphasize modesty, restrict open discussions about sexuality, and place significant value on fertility and reproduction, infertile couples often develop a particularly negative sexual self-concept. This negativity frequently manifests as heightened sexual anxiety, feelings of inadequacy and failure (especially linked to inability to conceive), diminished sexual confidence, reduced perceived sexual attractiveness, and difficulties in maintaining marital intimacy. Such negative perceptions can intensify psychological distress, marital conflict, and further hinder sexual and relational well-being^10–12^.

Self-concept plays a significant role in sexual performance. It refers to a person’s overall evaluation of their personality, including their grasping of sexual tendencies, known as sexual self-concept^8^. This concept is crucial for sexual health as it influences a person’s sexual desires and behaviors, which in turn can impact their mental and sexual well-being^9^. Achieving satisfying sexual experiences is essential for the success and stability of a relationship and family^13^. Therefore, counseling and educational interventions in this area can be beneficial and necessary^14^.

Therapeutic techniques for couples are highly valued in counseling because they focus on minimizing conflicts and enhancing communication within relationships^15^. This type of counseling is supportive and solution-oriented, focusing on identifying issues, proposing solutions, and ultimately promoting cognitive and behavioral changes within individuals. It encompasses all key components of interpersonal relationships^16^.

Multiple frameworks are available to assist healthcare providers in implementing an appropriate and effective strategy to address sexual concerns and problems, the most important of which is the Ex-PLISSIT model. This model provides a framework for developing and implementing interventions to help individuals maintain sexual relationships and enhance sexual satisfaction and performance throughout the reproductive cycle. The Ex-PLISSIT model includes permission stages, limited information, specific suggestions and therapy, and referral and follow-up. In the Ex-PLISSIT model, assessing and reflecting on all actions and interventions with the patient is essential^17,18^. Reviewing and reflecting on all patient interactions and interventions is crucial when using this model. Studies have shown that the implementation of this model can successfully decrease sexual issues in couples^19–21^. The Ex-PLISSIT model was selected to improve sexual self-concept due to its structured, stepwise approach (permission, limited information, specific suggestions, and intensive therapy), which addresses sexual concerns progressively. This model is particularly effective for infertile couples as it facilitates open communication, provides tailored education, and supports psychological adjustment, all of which are critical for enhancing sexual self-concept, as supported by prior research^22^.

Infertility is a crisis that affects individuals and society, causing disruptions in relationships. Utilizing counseling services based on the Ex_PLISSIT model can be beneficial in improving sexual performance. Due to the lack of research in this area, this study aims to explore the impact of couple-oriented counseling on infertile couples’ sexual self-concept following unsuccessful assisted reproductive methods at Fatimah Hospital in Hamadan City.

## Methods

### Study design and participants

This randomized controlled clinical trial was conducted from 2022 to 2023 at the infertility clinic of Fatemieh Hospital, Hamadan, Iran.

Participants and eligibility criteria Infertile couples were selected through convenience sampling after obtaining ethical approvals. Inclusion criteria were: willingness to participate, primary infertility, history of at least one failed assisted reproductive technique (e.g., IVF), infertility duration of 1–5 years, living together (no geographic separation), monogamy, women aged 18–40 years, men aged 18–60 years, residence in Hamadan, Persian language proficiency, both partners scoring 0–64 on the negative sexual self-concept subscale of the Multidimensional Sexual Self-Concept Questionnaire (higher scores indicating more negative self-concept), no psychiatric history, and no use of medications affecting sexual function.

Exclusion criteria included: missing multiple sessions, unexpected events during the study, withdrawal, only one partner participating, or natural conception during the trial.

### Sampeling

Sample size was calculated based on sexual satisfaction data from Malakouti et al.^21^ using α = 0.05, power = 0.90, M1 = 202.2, M2 = 182, SD1 = 16.14, SD2 = 24.17, and accounting for 30% attrition, resulting in 30 couples per group (total *N* = 60).

Randomization After obtaining written informed consent, eligible couples (*N* = 60) were randomly allocated to intervention (*n* = 30) or control (*n* = 30) groups using block randomization (six blocks of four: sequences ABB A, BBAA, ABAB, BABA, BAAB, ABBA). The allocation sequence was generated in advance and concealed in sealed, opaque envelopes.

### Intervention

The intervention group received four weekly, 60-minute, in-person couple-based counseling sessions (each couple separately, not group format), delivered by a trained research midwife following the Ex-PLISSIT model (Table 1).

**Fig. 1:**
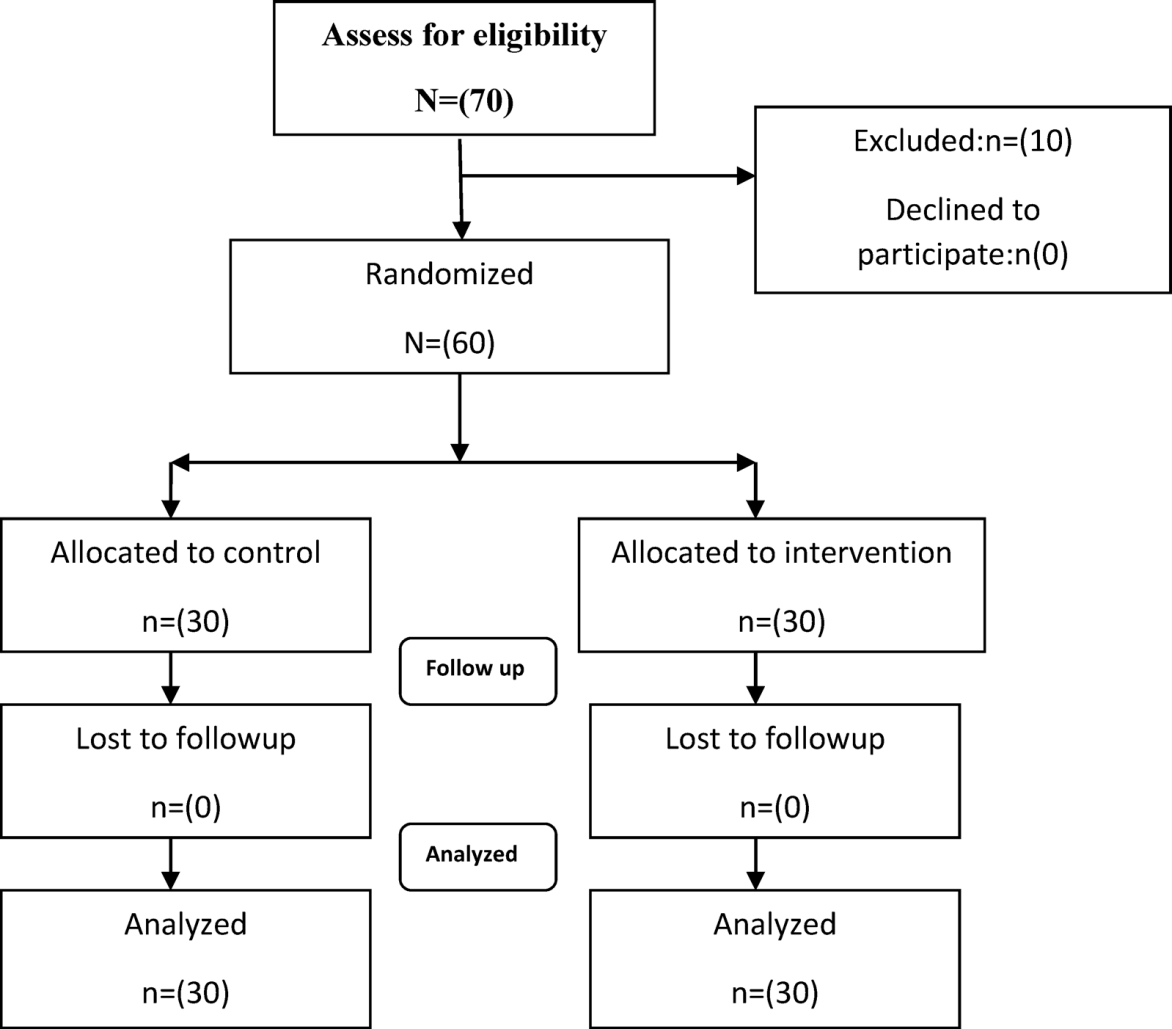
CONSORT flow diagram of the study process.

Figure 1 Flowchart of the randomized controlled clinical trial process to evaluate the effect of couple-based counseling using the Ex-PLISSIT model on the sexual satisfaction of infertile couples. This flowchart illustrates the steps of sample selection, random allocation, intervention, and outcome assessment.

Content was drawn from sexual counseling training, couple therapy principles, and the book “Couple Therapy” by Najafi and Shahbazi^23^, compiled into a pamphlet given to the intervention group after session 4. The control group received routine infertility care only. To ensure ethical standards, the control group received the educational pamphlet and session summary at study completion. Baseline assessments started on September 15, 2022, and post-intervention assessments occurred four weeks after the last session (October 13, 2022), in a private room at the hospital. Weekly phone follow-ups were conducted for feedback.

### Data collection tools

Data collection tools Data were collected using a demographic checklist and the Persian-validated Multidimensional Sexual Self-Concept Questionnaire (MSSCQ) by Ziaei et al. (2013)^22^. This 78-item tool uses a 5-point Likert scale (0 = not at all to 4 = completely applies). For this study, only the negative sexual self-concept subscale (max score 64) was analyzed. Cronbach’s alpha in this study was 0.94. The reliability of this questionnaire has been reported from 76.0% to 89.0% in different dimensions^24^. In the present study, the Cronbach’s alpha coefficient was calculated to be 0.94.

**Table 1:** Summary of Ex-PLISSIT couple-based counseling sessions.

Session	Ex-PLISSIT Stage	Duration	Key Content and Activities
1	Permission	60 min	Creating a safe, non-judgmental space; collecting sexual history using open-ended questions; reflecting participants’ statements to clarify ambiguities and build trust
2	Limited Information	60 min	Providing targeted education on sexual anatomy, physiology, sexual response cycle, definition and types of sexual self-concept, and the impact of negative self-concept on marital and fertility life; couples verbalized understanding and misconceptions were corrected
3	Specific Suggestions	60 min	Offering individualized suggestions based on identified issues (psychological factors, beliefs, behaviors affecting desire and satisfaction); content reflection to enhance self-awareness and provide tailored feedback
4	Intensive Therapy	60 min	Summarizing all sessions; reviewing changes in sexual self-concept; addressing remaining questions; final reflection and closure

### Data analysis

Quantitative data normality was assessed using the Shapiro-Wilk testDescriptive statistics (mean, SD, frequency) were used for demographic variables. Independent t-test or Mann-Whitney U test was applied for between-group comparisons of continuous variables, and chi-square or Fisher’s exact test for categorical variables. Paired t-test or Wilcoxon signed-rank test was used for within-group pre-post comparisons. Analysis of covariance (ANCOVA) was performed to compare post-intervention scores between groups while adjusting for baseline scores, smoking status, and male occupation. All analyses were conducted using SPSS version 26 with a significance level of *P* < 0.05.

## Results

The independent t-test results showed no statistically significant difference between the average age of men, average age of women, average duration of marriage, and average duration of infertility in the two control and intervention groups (*p* ≥ 0.05). The majority of women in the intervention group had a high school diploma, while in the control group, they had a diploma. Similarly, the majority of men in the control and intervention groups had a high school diploma; however, the results of the exact Fisher test showed no statistically significant difference between the two intervention and control groups in men (*p* = 0.562) and women (*p* = 0.945). On the other hand, men in the intervention group did not use tobacco, while 80% of the control group used tobacco. The results of the exact Fisher test showed a statistically significant difference between the two groups (*p* = 0.024). The majority of individuals in the intervention and control groups had common causes of infertility, 53% and 50%, respectively; however, this difference was not statistically significant (*p* = 0.836). Other demographic characteristics of the participating couples in the study are mentioned in Table 2.

**Table 2:** Demographic characteristics of the couples participating in the study in intervention and control groups.

Variables			Intervention grou Mean ± SD	Control group Mean ± SD	statistic	*P*-value
Men age (years)			35.40(5.82)	36.60(4.22)	0.91	0.365*
Women age (years)			31.30(5.88)	33.17(4.89)	1.34	0.187*
Duration of marriage (years)			6.97(2.03)	7.53(2.26)	1.02	0.310*
Duration of infertility (years)			4.03(1.16)	4.48(0.91)	-1.72	0.086*
Job (Women)		Employee	24(80)	27(90)	-	#0.472
		Housewife	6(20)	3(10)		
Women education		Cycle	12(40)	11(36.7)		
		Diploma	10(33.3)	12(40)	0.60	#0.945
		Bachelor’s degree	7(23.3)	6(20)		
		Master’s degree	1(3.3)	1(3.3)		
Job (Men)		Employee	8(26.7)	2(6.7)	4.32	#0.038
		Free	22(73.3)	28(93.3)		
Men education		Cycle	8(26.7)	11(36.7)		
		Diploma	13(43.3)	14(46.7)		
		Bachelor’s degree	8(26.7)	5(16.7)	2.14	#0.562
		Master’s degree	1(3.3)	0(0)		
Smoking(Men(		Yes	0(0)	6(20)	-	#0.024
		No	30(100)	24(80)		
economic situation		Low level	9(30)	14(46.7)		
		Average level	19(63.3)	16(53.3)	2.95	#0.198
		High level	2(6.7)	0(0)		
Causes of infertility		Female	7(23.3)	6(20)		
		Male	7(23.3)	9(30)	0.36	#0.836
		Both	16(53.3)	15(50)		

^*^ Independent t-test.

^#^Fisher exact test.

Table 3 displays the average scores of women’s self-concept before and after the intervention in both the intervention and control groups. Prior to the intervention, the average score for women in the intervention group was slightly higher at 21.50 (4.86) compared to the control group’s 19.87 (8.41), but this difference was not statistically significant (*p* = 0.361). Following the intervention, the average score for women in the intervention group decreased to 17.93 (6.03), while the control group’s average score increased to 21.83 (8.97), with a non-significant difference (*p* = 0.053). Conversely, there was a significant difference in the average scores of men’s self-concept post-intervention, with the intervention group scoring lower than the control group (*p* = 0.003).

**Table 3:** Comparison of the average sexual self-concept scores among women and men in both the intervention and control groups.

Variables		Intervention group Mean ± SD	Control group Mean ± SD	Statistic	*P*-value*
Women	Sxual self-concept scores(Before intervention)	21.50(4.86)	19.87(8.41)	-0.92	0.361
	Sexual self-concept scores(after intervention)	17.93(6.03)	21.83(8.97)	1.98	0.053
Men	Sexual self-concept scores(Before intervention)	20.83(5.38)	21.07(5.49)	0.17	0.868
	Sexual self-concept scores(after intervention)	17.10(5.76)	21.43(5.12)	3.08	0.003

^*^ T-test.

Results of the paired t-test showed a statistically significant difference in the average self-concept scores of women before and after the intervention in the intervention group (*p* = 0.009) as well as the control group (*p* = 0.003). In men, there was also a statistically significant difference in the average self-concept scores before and after the intervention in the intervention group (*p* = 0.001). However, this difference was not significant in the control group (*p* = 0.281) (Table 4).

**Table 4:** Comparison of the average self-concept scores in women and men within the intervention and control groups.

Variables		Sexual self-concept scores(Before intervention)	Sexual self-concept scores(after intervention)	T	*P*-value*
Women	Intervention grou Mean ± SD	21.50(4.86)	17.93(6.03)	3.27	0.009
	Control group Mean ± SD	19.87(8.41)	21.83(8.97)	-2.80	0.003
Men	Intervention grou Mean ± SD	20.83(5.38)	17.10(5.76)	4.40	0.001
	Control group Mean ± SD	21.07(5.49)	21.43(5.12)	-1.10	0.281

*Paired -test.

The post-intervention self-concept scores of women were compared to their pre-intervention scores. The results indicated that the average score in the intervention group was significantly lower than in the control group (*p* = 0.001). Likewise, for men, the average score in the intervention group was significantly lower than in the control group after adjusting for pre-intervention scores, smoking, and occupation (*p* = 0.001). The ANCOVA analysis revealed a significant effect of the pretest (F = 12.50, *p* = 0.002) and group (F = 17.39, *p* = 0.001) on the post-intervention sexual self-concept scores, indicating that baseline scores and group assignment were key factors influencing the outcomes.

The analysis of covariance (ANCOVA) results, as shown in Table 5, highlight the impact of the Ex-PLISSIT counseling on post-intervention sexual self-concept scores among infertile couples. The adjusted mean score for women in the intervention group was 17.24 (SD = 4.98), significantly lower than the control group’s 22.53 (SD = 4.98) (F = 16.80, *p* = 0.001), indicating a notable improvement. For men, the intervention group’s adjusted mean score was 17.45 (SD = 3.23) compared to 21.09 (SD = 3.23) in the control group (F = 17.39, *p* = 0.001), also showing significant enhancement. The mean difference column in Table 5 represents the absolute difference in adjusted post-intervention sexual self-concept scores between the intervention and control groups (calculated as control mean minus intervention mean), along with its 95% confidence interval (CI), which quantifies the precision of this estimate and indicates the range within which the true difference is likely to lie. The standardized mean difference (also known as Cohen’s d) provides a measure of the effect size, standardizing the mean difference by the pooled standard deviation to allow for comparison across studies; values around 0.2 indicate a small effect, around 0.5 a medium effect, and around 0.8 or greater a large effect, as noted in the table footnote. In this study, the standardized mean differences (1.06 for women and 1.13 for men) suggest large effect sizes, underscoring the substantial clinical impact of the intervention. Covariate analysis revealed that pretest scores (F = 12.50, *p* = 0.002) and group assignment (F = 17.39, *p* = 0.001) significantly influenced the outcomes, while smoking (F = 3.45, *p* = 0.067) and occupation (F = 2.89, *p* = 0.094) had no significant effect. These findings confirm the effectiveness of the intervention in reducing negative sexual self-concept, particularly when adjusted for baseline and demographic factors (Table 5).

**Table 5:** Comprehensive ANCOVA^*^ Results for Post-Intervention Sexual Self-Concept.

Variable		After intervention Mean ± SD	Mean Differences (95%CI)	Standardized mean **differences (95%CI)	F	*p*-value
Women	Intervention	17.24 (4.98)	5.29 (2.72 to 7.86)	1.06 (0.47 to 1.65)	16.80	0.001
	Control	22.53 (4.98)				
Men	Intervention	17.45 (3.23)	3.64 (1.97 to 5.31)	1.13 (0.54 to 1.71)	17.39	0.001
	Control	21.09 (3.23)				

*Adjusted for pre-test scores, smoking, and occupation.

**A value around 0.2 indicates a small effect, around 0.5 a medium effect, and around 0.8 or greater a large effect.

## Discussion

The present randomized controlled clinical trial aimed to determine the effect of couple-based counseling using the Ex-PLISSIT model on the sexual self-concept of infertile couples.

The most important finding indicates that couple-based counseling significantly reduces negative sexual self-concept in infertile couples. Among the dimensions assessed with the Modified Multidimensional Sexual Self-Concept Questionnaire (MSSCQ), the situational sexual self-concept dimension showed the least response to the intervention (*p* > 0.05, data not shown). This may be due to its dependence on external factors such as cultural taboos, immediate stressors from infertility treatments, and the limited number of sessions, which were insufficient to deeply address context-specific concerns. In the Iranian community studied, cultural sensitivities and reluctance to openly discuss situational sexual issues—coupled with the couples’ primary focus on fertility outcomes—likely limited the intervention’s effectiveness on this dimension^10^. This suggests the need for extended or more culturally tailored interventions in future research.

This is the first study in Iran to examine sexual self-concept in infertile couples using the Ex-PLISSIT model. Given the scarcity of research on infertile couples, we reviewed global studies showing that couple-based or Ex-PLISSIT counseling reduces sexual issues in various chronic conditions^11,25–29^.

Gordani et al. (2021) found that mood regulation training significantly improved general and sexual self-concept in infertile women, reducing negative perceptions (e.g., sexual anxiety, depression, monitoring, and fear of intercourse) and increasing positive aspects^30^. Although their study was group-based and limited to women, while ours was couple-centered, both showed beneficial effects on sexual self-concept. The couple format in our study allowed more comfortable expression of personal questions and problems, potentially reducing fear of judgment or embarrassment that might occur in group settings.

Lotfollahi et al. (2021) reported higher negative and lower positive sexual self-concept in infertile versus fertile women, recommending routine screening and counseling at infertility centers^11^. Ziaei et al. (2017) demonstrated that increasing positive sexual self-concept and decreasing negative self-concept enhances sexual function in reproductive-age women, suggesting counseling as an effective method for sexual health and family stability^31^. These findings are consistent with our results, including no significant change in the control group.

Yazdi et al. (2020) showed that sexual self-concept counseling via social networks improved sexual self-concept and couple relationships in infertile women, with reductions in negative aspects and no change in situational self-concept^6^. Our in-person, couple-based approach extends this by involving both partners, which may strengthen outcomes in a face-to-face setting.

Agustus et al. (2017) highlighted insufficient sexual knowledge and distorted mental images as key factors in sexual disorders among infertile women^32^. Through couple-centered counseling, we reduced negative sexual self-concept, including monitored aspects like sexual depression.

One strength of this study is the couple-centered design, which promotes communication, spouse involvement, and problem resolution. Previous research indicates that Iranian society is reluctant to discuss sexual problems openly due to cultural considerations, yet individuals are interested in general sexual topics^10,32^. The presence of both partners facilitates expression and may be more effective than individual approaches^32^.

Limitations and strengths Limitations include questionnaire fatigue, recruitment challenges due to personal concerns and cultural views of sexual counseling as private, and short-term follow-up (four weeks with weekly calls), which may not capture long-term effects. Future studies should include extended follow-ups (e.g., 3, 6, 12 months). Strengths include the randomized controlled design, validated Persian MSSCQ, couple-based delivery, and confounder adjustment in ANCOVA. This model is recommended as a cost-effective, simple method for improving sexual health in infertile couples.

## Conclusion

Couple-oriented counseling with the Ex-PLISSIT model improves negative sexual self-concept in infertile couples. Routine assessment of sexual self-concept and performance at infertility centers, with appropriate counseling and support, is advised for professionals in gynecology, obstetrics, psychology, and related fields.

## Data Availability

The datasets generated during the current study are not publicly available due to confidentiality agreements with the participants. However, they are available from the corresponding author on reasonable request and with appropriate ethical approvals.
